# Initial surveillance of suspected undesirable drug reactions to some Artemisinin-based combination therapies (ACTs) in Lagos, Nigeria

**DOI:** 10.1186/1475-2875-9-S2-P1

**Published:** 2010-10-20

**Authors:** Bamgboye M Afolabi, Antonia Dunkwu, A C Oparah

**Affiliations:** 1 Health, Environment and Development Foundation, 34 Montgomery Road, Yaba, Lagos, Nigeria; 2 National Agency for Food and Drugs Administration and Control, Oshodi, Lagos, Nigeria; 3 Faculty of Pharmacy, University of Benin, Benin City, Edo State, Nigeria

## Background

The exacerbation of malaria in sub-Saharan Africa towards the end of the 20th century accrued from amplified resistance to chloroquine and sulfadoxine-pyrimethamine (SP) the most affordable antimalaria medicines [1]. Contact with substandard antimalarial medicines probably aggravated this development [2-4]. Pressure from malaria scientists triggered extensive acceptance and implementation of artemisinin-based combination therapies (ACTs) by endemic country governments and donors [5]. All medicines carry some risk of harm and it is important to monitor their intended and unwanted effects for good evidence upon which to base an assessment of risk versus effectiveness or risk versus benefit. Furthermore, particularly with new medicines, the early identification of unexpected adverse reactions and their risk factors is essential, so that the medicines can be used in an informed manner with the least chance of harm. This is the role of pharmacovigilance (PV). Information gathered during pharmacovigilance may also assist in selecting the most appropriate medicine for future use. The objectives of this study were to document the suspected undesirable but common adverse drug reactions (SADRs) of patients using various Artemisinin-based Combination therapies (ACTs) in Lagos, (Nigeria) and to document the involvement of community pharmacists in the detection and reporting of these ADRs.

## Materials and methods

A semi-structured questionnaire was served to each of the 235 randomly selected community pharmacists/respondents in urban and semi-urban Lagos metropolis between May and July 2008 out of which 201 (83.8%) responded.

## Results

Nine hundred and sixty one reports of SADR to Artesunate-Amodiaquine (AA) combination were reported followed by 642 to dihydro-artemisinin-SP combination, 521 to Artesunate-Mefloquine combination, 401 to Artemisinin monotherapies and 401 to Artemeter-Lumefantrin combination (Table 1). When Artesunate Amodiaquine was compared with Artemeter Lumefantrin, there were significant differences in the proportion of patients with weakness and fatigue (102, 37.8%; 32, 11.9%; OR 4.52; p<0.0001), dizziness (93, 38.0%; 32, 12.1%; OR 4.07; p<0.0001), headache (51, 27.9%; 25, 13.7%; OR 2.44; p<0.0001), insomnia (46, 36.5%; 13, 10.3%; OR 5.00; p<0.0001), abdominal discomfort (52, 28.5%; 30, 17.0%; OR 1.6; p<0.05) and depression (14, 44.1%; 3, 8.8%; OR 8.16; p<0.0001).

**Table 1:** Proportion of patients with suspected adverse drug reactions (SADRs) to various Artemisinin-based combination therapies (ACT) in Lagos (2008).

SADRs	Artemisinin Monotherapy (number of reports=410)	Artesunate-Mefloquine (number of reports=521)	Artesunate-Amodiaquine (number of reports=961)	Artemeter-Lumefantrine (number of reports=401)	Dihydro artemisinin/SP (number of reports=642)
Anorexia	19	25	52	14	33
Tightening of chest	7	17	26	13	9
Rashes	10	21	28	20	55
Pruritis	6	*22*	39	16	28
Dizziness	33	48	93	32	39
Restlessness	35	35	79	24	29
Headache	39	31	51	25	37
General body pain	19	24	43	16	18
Weakness/fatigue	43	47	102	32	46
Vomiting	5	11	15	5	20
Abdominal discomfort	37	25	52	30	32
Insomnia	17	21	46	13	29
Swollen/blistered lips	9	6	10	11	46
Hallucination	3	17	16	9	14

## Conclusions

Suspected adverse drug reactions especially to AA are factors that limit its use as an effective ACT which has unpalatable reactions to the patient. The confidence of end users of this particular ACT wanes with the development of suspected but constant mild, moderate to severe adverse drug reactions.

## References

[B1] Gomes M Wayling S Pang L Interventions to improve the use of antimalarials in south-east Asia: an overview Bull World Health Organ 1998 76 9 19 9763718 PMC2305573

[B2] Minzi OMS Moshi MJ Hipolite D Massele AY Tomson G Evaluation of the quality of amodiaquine and sulphadoxine/pyrimethamine tablets sold by private wholesale pharmacies in Dar Es Salaam Tanzania Journal of Clinical Pharmacy and Therapeutics 2003 28 117 122 10.1046/j.1365-2710.2003.00470.x 12713608

[B3] Amin AA Snow RW Kokwaro GO The quality of sulphadoxinepyrimethamine and amodiaquine products in the Kenyan retail sector Journal of Clinical Pharmacy and Therapeutics 2005 30 559 565 10.1111/j.1365-2710.2005.00685.x 16336288 PMC3521059

[B4] Basco LK Molecular epidemiology of malaria in Cameroon. XIX. Quality of antimalarial drugs used for self-medication Am J Trop Med Hyg 2004 70 245 250 15031511

[B5] Attaran A Barnes KI Curtis C D'Alessandro U Fanello CI WHO, the Global Fund and medical malpractice in malaria treatment The Lancet 363(9404) 2004 237 240 10.1016/S0140-6736(03)15330-5 14738799

